# Endoscopic Study of Ethmoidal Canals in Cadavers, Including a Histological Analysis of Their Contents

**DOI:** 10.1055/s-0043-1767805

**Published:** 2023-09-26

**Authors:** Alexandre Wady Debes Felippu, Thiago Picolli Morsch, André Wady Debes Felippu, Filippo Cascio, Claudia Regina Gomes Cardim Mendes de Oliveira, Alexandre Felippu, Richard Louis Voegels

**Affiliations:** 1 Department of Otorhinolaryngology, Hospital das Clínicas, Faculdade de Medicina, Universidade de São Paulo, SP, Brazil; 2 Instituto Felippu de Otorrinolaringologia, São Paulo, SP, Brazil; 3 Department of Otorhinolaryngology, Azienda Ospedaliera Papardo, Messina, Italy; 4 Institute of Orthopedics, Hospital das Clínicas, Faculdade de Medicina, Universidade de São Paulo, SP, Brazil

**Keywords:** ethmoid sinus, ophthalmic artery, dissection, ethmoid bone, skull base

## Abstract

**Introduction** The advent of the endoscope has enabled the use of the endonasal approach for a variety of diseases. Studying the ethmoidal canals is important for surgeries of the paranasal sinuses and the anterior base of the skull.

**Objective** To investigate the ethmoidal canals and evaluate their structure, the presence of vessels and nerves, their location, and to perform an anatomopathological study of their contents.

**Methods** We evaluated 20 cadavers (20 left and 20 right nasal cavities) through endoscopic dissection of the anterior base of the skull and exposure of the medial periorbita and dura mater; then, the ethmoidal canals were located and measured in relation to the anterior wall of the sphenoid sinus and between the ethmoidal canals, followed by removal of their content for histological analysis.

**Results** Vessels were present in 75% of the left anterior ethmoidal canals, 70% of the left posterior ethmoidal canals, 75% of the left middle ethmoidal canals, 85% of the right anterior ethmoid canals, and 64.5% of the right posterior ethmoid canals; 50% of the right middle ethmoidal canals contained one vessel.

**Conclusion** The ethmoidal canal does not necessarily contain an ethmoidal artery. Studies with a larger sample should be performed to quantify the correct proportion of arteries and ethmoidal canals.

## Introduction

The advent of the endoscope has enabled the use of the endonasal approach in several types of surgeries other than those to treat diseases of this region: it also became an access route for surgery in the orbit, the anterior base of the skull, and the middle and posterior fossae. As a result, understanding endonasal anatomy has become increasingly important. 1 2 3

Vascularization of the nose occurs mainly via the sphenopalatine and ethmoidal arteries. The former is part of the external carotid system, while the latter, of the internal carotid system. Ethmoidal arteries, the object of the present study, share a part of the vascularization of the nasal cavities, and they have been involved in the etiology of certain cases of epistaxis, particularly in the posterior nasal region of elderly patients. Failure to identify the ethmoidal arteries can result in inadvertent injury to these structures, which, in some cases, can cause intraorbital hematoma, and could eventually progress to amaurosis. 4 5 6

Caliot et al. 4 (1995) studied 100 dried skulls, and, while analyzing the ethmoidal foramina and the ethmoidal arteries, found 2 foramina in 2% of the cases, 2 ethmoidal foramina in 80% of the cases, 3, in 16.5% of the cases, and 4, in 0.5% of the cases, concluding that there may be more than two ethmoidal arteries in the nasal cavity. However, the study did not assess the content of these foramina and could not prove the existence of additional arteries within the canals.

Han et al. 7 (2008) studied 24 cadavers (48 sides) via an external Lynch incision to endoscopically determine the location of the ethmoidal arteries. The anterior ethmoidal artery was absent in 2 nasal cavities, while the posterior artery was only absent in 1 of the 48 cavities. Such results demonstrate the existence of divergences within the literature on the subject. 7

Differing again from previous information, in 2014, Wang et al., 5 under endoscopic view, studied the possibility of the presence of a middle ethmoidal artery in 22 cadavers, and a third ethmoidal artery was identified in 31.8% of the sides studied.

Gras-Cabrerizo et al. 8 (2022) performed a dissection study of 17 cadavers (34 nasal cavities). They reported the presence of accessory ethmoidal canals in 4 cases (12%). In two canals, the presence of an artery was observed, in one canal, the presence of only 1 nerve, and in one of the canals, no structure was identified. In their research, the authors 8 did not perform a histological analysis of the content of the canals.

In another tomographic study, Monjas-Cánovas et al. 9 (2011) only located posterior ethmoidal arteries in 35% of the nasal cavities, which, again, is in disagreement with preexisting data. In a brief summary of the literature, the authors acknowledged the actual problem:

“The radiological anatomy of the ethmoidal arteries has been widely studied. Most works published refer to CT observations of the anterior and posterior ethmoidal canals themselves. However, due to the technical limitations of the scanner, their arterial contents and the intra-orbital and intra-cranial segments have hardly been referenced”. 9

The content of the ethmoidal canals has been studied in very few articles, which may lead one to conclude that the supposed posterior and middle ethmoidal arteries are not actually arteries, or that there are other arteries that tomography has been unable to identify.

The main objective of the present study is to expand the knowledge on the ethmoidal canals through a histological analysis of their content, which will enable better surgical planning and may reduce the rate of complications.

## Methods

The present study was carried out at our university's dissection lab. After approval by the Institutional Ethics in Research Committee (under number 620838 16.8.0000.0065), 20 fresh cadavers were dissected and analyzed.

We excluded from the sample cadavers that presented absence of orbital content, signs of facial trauma, previous epistaxis, or any endonasal procedure.

Dissection was performed in the following steps: incision to the septal mucosa; detachment of the septal mucosa; removal of septal cartilage and perpendicular plate of the ethmoid, preserving 1.5 cm cranially. This was followed by dissection of the paranasal sinuses, ethmoidectomy via the centripetal technique, maxillary sinusotomy, frontal sinusotomy, identification of the ethmoidal foramina and canals, and exposure of the medial periorbita. The dura mater of the anterior base of the skull was exposed, with emphasis on preserving the contents of the ethmoidal canals. 10

Then, we performed an endoscopic analysis of the ethmoidal canals which were divided into: bone canals (BCs), when their contents were covered by bone; intracranial canals (ICs), when the structures were not visible at the base of the skull; and dehiscent (D), when structures were exposed.

**Fig. 1:**
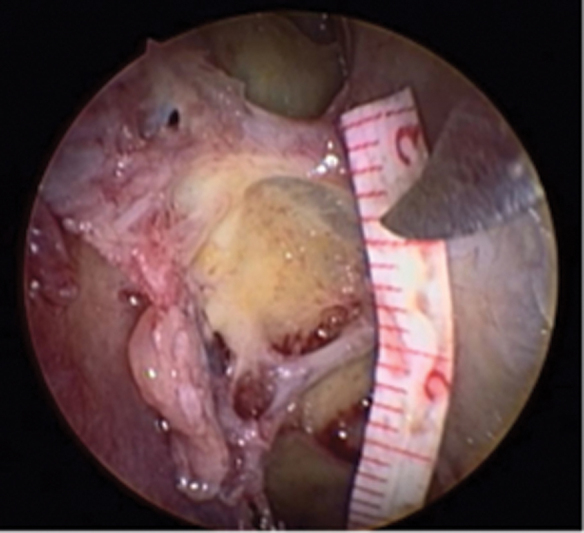
Left nasal cavity; 30° endoscope; exposure of the anterior base of the skull andmedial periorbital wall; visualization of anterior and posterior ethmoidal canals; measurement between the ethmoidal canals.

**Fig. 2:**
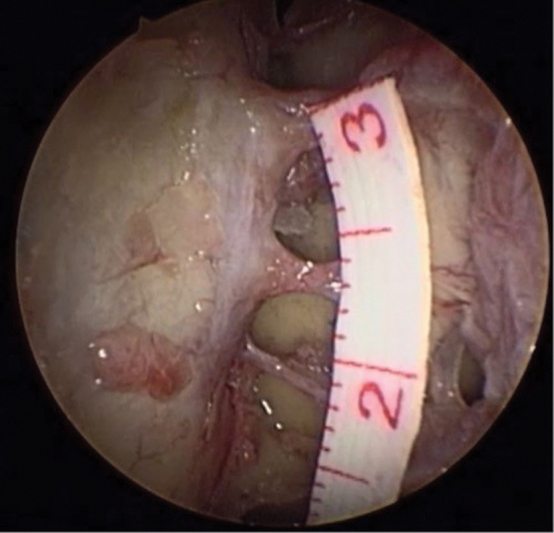
Right nasal cavity; 30° endoscope; exposure of the anterior base of the skull and medial periorbital wall; visualization of anterior, middle, and posterior ethmoidal arteries; measurement between the ethmoidal canals.

The dissection procedure followed with measurement of the ethmoidal canals in relation to the anterior wall of the sphenoid sinus and between the ethmoidal canals ( Figs. 1 and 2 ). It ended with the removal of the contents for histological analysis. The resected fragments were fixed in 10% formalin and buffered for up to 24 hours.

**Fig. 3:**
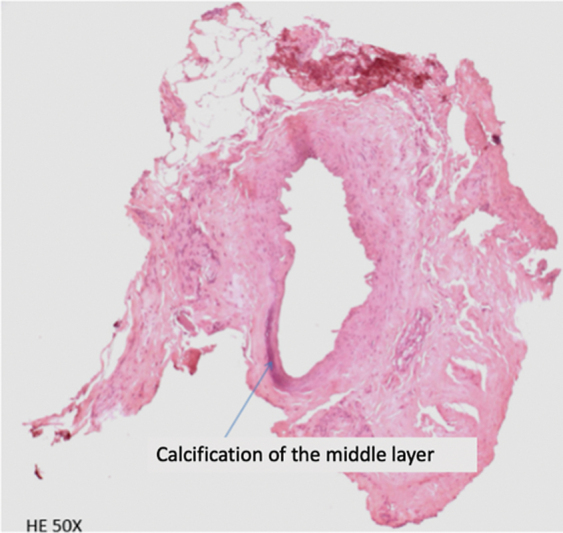
Histological assessment of the content of the ethmoidal canal, performed with hematoxylin and eosin, demonstrating an artery with calcification of the middle layer.

**Fig. 4:**
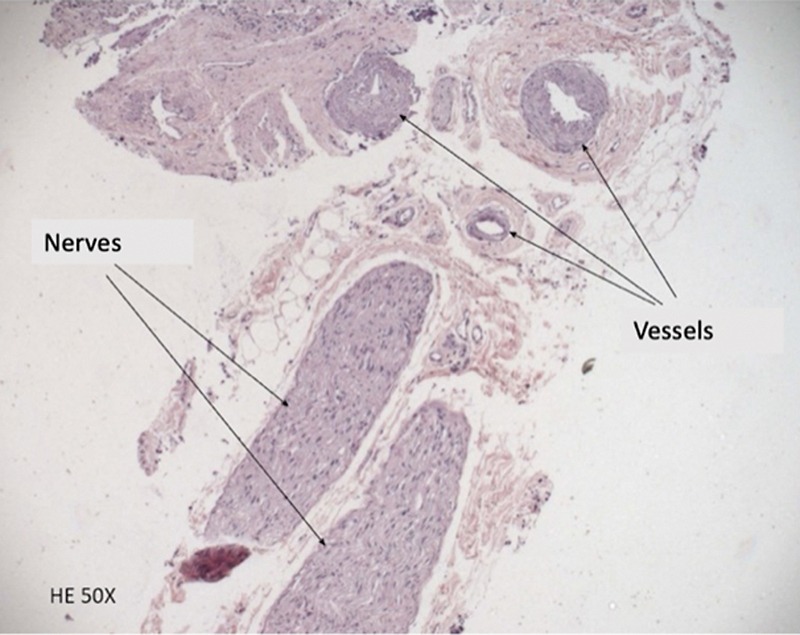
Histological assessment of the content of the ethmoidal canal, performed with hematoxylin and eosin, demonstrating nerves and blood vessels.

A histological evaluation was performed to identify whether or not the interior of the canals contained the following variables: vascular structure; neural structure; and dense connective tissue ( Figs. 3 and 4 ).

Technical processing was carried out in the automated tissue processor in the following steps: sagittal cuts with a thickness of 5 μm through the microtome of the material embedded in paraffin; staining by the hematoxylin and eosin procedure; and histological evaluation by pathology-otolaryngology double observation.

## Results

Regarding the cadavers surveyed, 14 (70%) were male and 6 (30%) were female, with a mean age was of 64 (median: 63.5) years, mean body weight of 67.35 Kg, and mean height of 168 cm, (median: 167 cm).

Regarding the distances between the ethmoidal canals and of the canals in relation to the anterior wall of the sphenoid sinus, the findings were: left anterior ethmoidal canal (LAEC) in relation to the left posterior ethmoidal canal (LPEC) – mean of 1.45 cm; LPEC in relation to the sphenoid (S) – mean of 0.65 cm; right anterior ethmoidal canal (RAEC) in relation to the right posterior ethmoidal canal (RPEC) – mean of 1.41 cm; RPEC–S – mean 0.78 cm; LAEC in relation to the left middle ethmoidal canal (LMEC) – mean of 0.93cm; and RAEC in relation to the right middle ethmoidal canal (RMEC) – mean of 1.00cm ( Table1 ).

**Table 1:** Distances between ethmoidal canals and from them to the anterior wall of the sphenoid sinus

Distance	Mean (cm)
LAEC–LPEC	1.45
LPEC–S	0.65
RAEC–RPEC	1.41
RPEC–S	0.78
LAEC–LMEC	0.93
RAEC–RMEC	1.00
** General mean**	**1.04**

Abbreviations: LAEC, left anterior ethmoidal canal; LMEC, left middle ethmoidal canal; LPEC, left posterior ethmoidal canal; RAEC, right anterior ethmoidal canal; RMEC, right middle ethmoidal canal; RPEC, right posterior ethmoidal canal; S, sphenoid.

Regarding the content of the canals, vessels were present in 75% of the LAECs, 70% of the LPECs, 75% of the LMECs, 85% of the RAEC, and 64.5% of the RPECs; 50% of the RMECs contained 1 vessel ( Table 2 ).

**Table 2:** Presence or absence of vessels inside each ethmoidal canal

Canal			
	No vessel – n (%)	1 vessel – n (%)	> 1 vessel – n (%)
LAEC	5 (25%)	9 (45%)	6 (30%)
LPEC	6 (30%)	9 (45%)	5 (25%)
LMEC	1 (25%)	2 (50%)	1 (25%)
RAEC	3 (15%)	10 (50%)	7 (35%)
RPEC	7 (35%)	10 (50%)	3 (15%)
RMEC	1 (50%)	1 (50%)	0 (0.0%)

Abbreviations: LAEC, left anterior ethmoidal canal; LMEC, left middle ethmoidal canal; LPEC, left posterior ethmoidal canal; RAEC, right anterior ethmoidal canal; RMEC, right middle ethmoidal canal; RPEC, right posterior ethmoidal canal.

We found anterior and posterior ethmoidal canals bilaterally in all cadavers; 4 middle ethmoidal canals (20%) were found on the left side, and 2 middle ethmoidal canals (10%) were found on the right side.

Analyzing the presence of nerves inside the canals, we observed that they were present in 80% of the LAECs, 70% of the LPECs, 25% of the LMECs, 80% of the RAECs, 55% of the RPECs, and 50% of the RMECs ( Table 3 ).

**Table 3:** Presence or absence of nerves inside each ethmoidal canal

Canal		
	Without nerve – n (%)	With nerve – n (%)
LAEC	4 (20%)	16 (80%)
LPEC	6 (30%)	14 (70%)
LMEC	3 (75%)	1 (25%)
RAEC	4 (20%)	16 (80%)
RPEC	9 (45%)	11 (55%)
RMEC	1 (50%)	1 (50%)

Abbreviations: LAEC, left anterior ethmoidal canal; LMEC, left middle ethmoidal canal; LPEC, left posterior ethmoidal canal; RAEC, right anterior ethmoidal canal; RMEC, right middle ethmoidal canal; RPEC, right posterior ethmoidal canal.

In the endoscopic analysis of the ethmoidal canals, 85% of the LAECs were found in a bone canal (BC), none were IC, and 15% were dehiscent (D); 75% of the LPECs were in a BC, 15% were IC, and 10%, D; 75% of the LMECs were in a BC, 25% were IC, and none were D; 80% of the RAEC were in a BC, none were IC, and 20%, D; 75% of the RPECs were in a BC, 15% were CI, and 10%, D 10%; and 100% of the RMECs in CO.

## Discussion

The present is the first study that includes removal of the content of the ethmoidal canal and an anatomopathological evaluation. Although the number of studies on ethmoidal arteries has increased in recent years, 8 9 11 few have explored the content and performed a histological assessment of the interior of the ethmoidal canal. Gras-Cabrerizo et al. 8 (2022) addressed the issue that ethmoidal canals may not necessarily contain arteries, but there was no histological analysis of the sample.

Ethmoidal arteries present great anatomical variability, not only in terms of their distance from the frontal sinus and anterior wall of the sphenoid, but also in terms of their relationship with the base of the skull. This proximity to the anterior base of the skull has great relevance to surgery in this region, but few studies 9 12 address this issue.

In the literature, anatomical knowledge of the posterior ethmoidal artery (PEA) and middle ethmoidal artery (MEA) is still far from ideal. Unlike the anterior ethmoidal artery (AEA), the relationship between the MEA and PEA with the ethmoidal lamellae or other types of anatomical repair has been little discussed. Data on prevalence, trajectory, blood flow, and distribution are scarce and limited. There are few studies that quantify the size of the structures, information that would be essential for epistaxis procedures, endoscopic surgical interventions in the anterior base of the skull, and orbital surgeries. The PEA and MEA have been described as thinner than the AEA; however, there is a lack of studies that specify the exact size and difference in caliber regarding them. 8 13 14 15 16 17 18 19

In the sample of the present study, four middle ethmoidal canals were found on the left, and two, on the right. Of these six canals, only four contained a blood vessel. The others contained only connective tissue. Despite the small sample, the results showed few middle ethmoidal canals with blood vessels. This encouraged an understanding that MEAs may be less relevant in surgery when compared to AEAs and PEAs. However, MEAs cannot be neglected, as they nourish a portion of dura mater of the anterior base of the skull and, in refractory epistaxis, they can acquire relevance. Because of the small sample, the data cannot be considered statistically relevant, but it is in agreement with the existing literature on the subject. We have found no studies proving that the MEA and PEA could not be one of the causes of failure in severe epistaxis surgeries. Therefore, further research is suggested to elucidate this issue.

The present study explored the presence of nerves inside the ethmoidal canals, but the authors found no practical relevance regarding this subject.

The nomenclature of certain structures in this region still lacks consistency. The MEAs have been eventually referred to as *supernumerary ethmoidal arteries* or *accessory ethmoidal arteries* , leading to miscommunication and imprecision. Moreover, regarding the nomenclature to define the relationship of the arteries with the base of the skull, in a study carried out in Malaysia by Raiagopalan et al. (2019), the distance was referred to as *short mesentery* (close to the base of the skull) and *long mesentery* (dehiscent), making it difficult to standardize the nomenclature.

Therefore, standardization of the nomenclature *middle* or *supernumerary ethmoidal arteries* is suggested, which is relevant to understand the anatomy, as well as standardization of nomenclature regarding the relationship between ethmoidal canals and the base of the skull.

To measure the ethmoidal arteries, some authors 7 22 23 24 have used the optic nerve as a parameter. The anterior wall of the sphenoid sinus, however, is believed to be a more adequate anatomical reference, since it is a structure that is perpendicular in relation to the position of the endoscope and parallel to the ethmoidal arteries, facilitating measurement.

As herein reported, most ethmoidal arteries are covered by a BC, which can make sinus surgery safer, but can also lead to a more complex cauterization. When a D ethmoidal artery is found, the reasoning becomes the opposite: although the risk of inadvertent injury increases, ligation of the artery becomes less laborious.

Surgeons often identify the ethmoidal canal on CT scans and label it the ethmoidal artery. However, we consider that the nomenclature *ethmoidal artery* ideally should only be used when there is certainty regarding the presence of a vessel inside the canal. Such care was not observed in the articles studied, since the researchers implied that arteries exist in the ethmoidal canals, which is not always true.

Studies 2 13 19 23 25 suggest that there are anatomical, epidemiological, and histological differences regarding the contents of the ethmoidal canals. However, more extensive and robust research is needed to reach more accurate conclusions.

In view of the number of failures in refractory bleeding surgery, the MEA and PEA should not be excluded from routine investigations of epistaxis, and should play a relevant role in the planning of skull base surgery.

We also considered that the identification of ethmoidal canals cannot be conflated with that of ethmoidal arteries, considering that many ethmoid canals do not contain an artery.

Future studies with larger samples should be performed to gain a better understanding of ethmoidal arteries and canals.

## Conclusion

Based on the present study, the ethmoidal canals do not necessarily contain an ethmoidal artery.

Studies with larger samples should be performed to quantify the correct proportion of arteries and ethmoidal canals.
